# Supplementary material for: Neutralizing antibodies after the third COVID-19 vaccination in healthcare workers with or without breakthrough infection
Source: Commun Med (Lond). 2024 Feb 23;4:28. doi: 10.1038/s43856-024-00457-3 (PMC10891120; doi:10.1038/s43856-024-00457-3)
Supplement: Supplementary file 2 — Supplementary Information [file 43856_2024_457_MOESM2_ESM.pdf]

Neutralizing antibodies after the third COVID-19 vaccination in healthcare workers with or without breakthrough infection

Supplementary information

Supplementary table 1. Number of collected serum samples and their timing in relation to vaccine doses

		Short dose interval 2x BNT162b2	Long dose interval 2x COVID-19 vaccine	Long dose interval 2x BNT162b2	Long dose interval 2x mRNA-1273	Long dose interval ChAdOx + BNT162b2 / mRNA-1273
Pre (days)	N	-	119/202	39	39	41
	mean	-	-2.0	-1.8	-0.8	-3.2
	range	-	-38–6	-22–6	-8–0	-38–6
1D3wk (weeks)	N	-	180/202	54	65	61
	mean	-	3.3	3.2	3.3	3.5
	range	-	2.4–5.3	2.9–4.3	2.7–5.0	2.4–5.3
1D3mo (months)	N	-	172/202	51	56	65
	mean	-	2.7	2.7	2.7	2.7
	range	-	1.9–3.8	1.9–3.5	2.5–3.8	2.4–2.9
2D3wk (weeks)	N	-	181/202	58	65	59
	mean	-	3.6	3.5	3.9	3.2
	range	-	1.9–6.6	1.9–6.4	2.6–6.6	2.0–4.9
2D3mo (months)	N	-	187/202	58	64	65
	mean	-	3.2	3.1	3.2	3.2
	range	-	1.9–4.2	1.9–4.1	2.5–3.9	2.9–4.2
2D6mo (months)	N	-	145/202	46	41	58
	mean	-	5.8	5.7	5.5	6.1
	range	-	4.5–7.0	4.5–7.0	4.5–7.0	5.7–6.8
2D8mo (months)	N	224/230	–	–	–	–
	mean	7.6	–	–	–	–
	range	4.6–12.4	–	–	–	–
3D3wk (weeks)	N	222/230	165/202	51	55	59
	mean	3.8	3.8	3.9	3.5	3.9
	range	1.7–9.6	1.6–7.1	1.6–7.1	2.7–5.9	2.6–6.0
3D3mo (months)	N	217/230	176/202	54	61	61
	mean	3.3	3.3	3.3	3.3	3.2
	range	2.6.–4.7	2.3–4.7	2.3–4.7	2.3–4.7	2.4–4.6
3D6mo (months)	N	224/230	156/202	38	58	60
	mean	6.3	6.1	6.2	6.1	6.2
	range	5.1–7.9	4.8–7.7	4.8–7.7	4.9-7.7	5.0–7.5
3D9mo (months)	N	224/230	24/202	7	1	16
	mean	9.2	9.1	8.9	8.3	9.2
	range	8.3–10.5	8.1–9.8	8.1–9.4	–	8.4-9.8

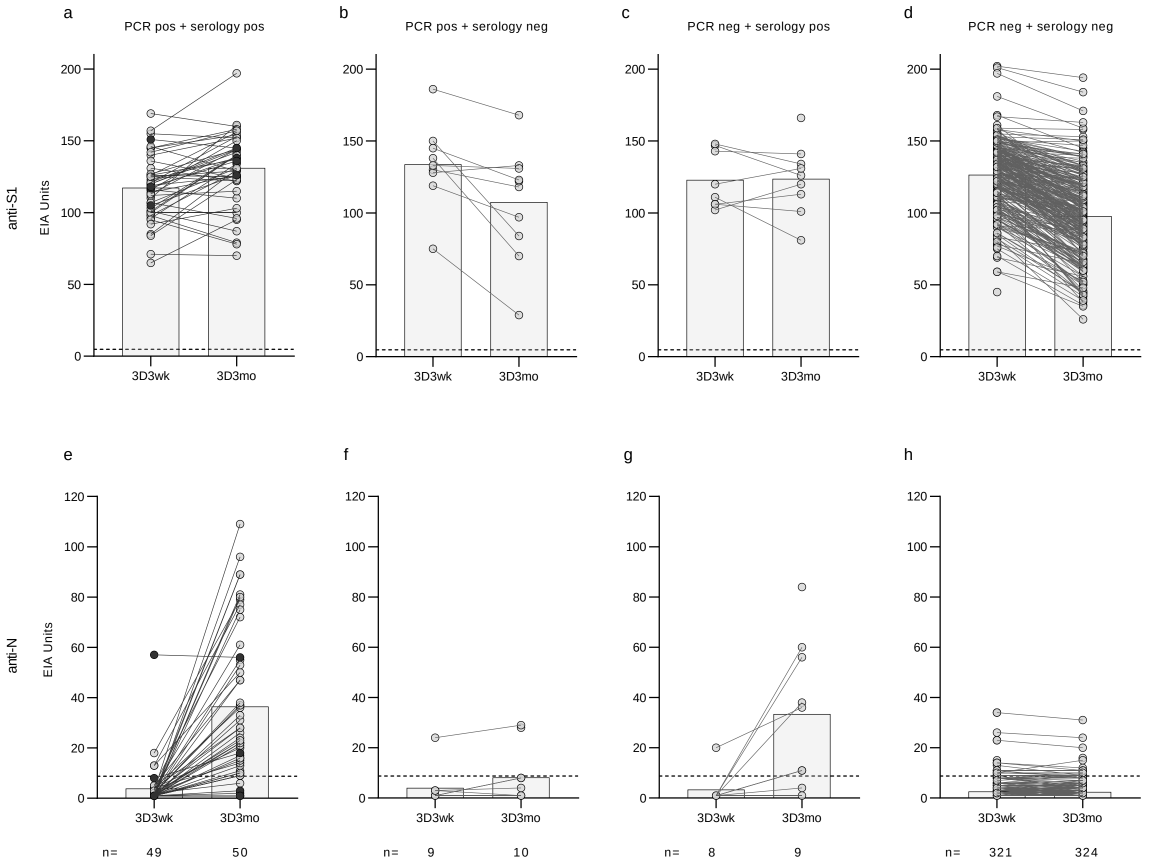

Supplementary figure 1. Changes in SARS-CoV-2 anti-S1 and anti-N antibodies from three week to three months post third vaccine dose grouped by PCR test or antigen test positivity and serologically confirmed infection. Thrice vaccinated HCWs were grouped into four groups (PCR pos/neg and serology pos/neg) based on their PCR or antigen test positivity three weeks to three months post third vaccine dose. Four vaccinees with a PCR positive test between third dose and three weeks after third dose were included in the PCR pos –group. Prevaccination infected vaccinees were marked with black dots. SARS-CoV-2 **a-d** S1-specific and **e-h** N-specific antibody responses of each group and the number of samples in each time point are indicated.

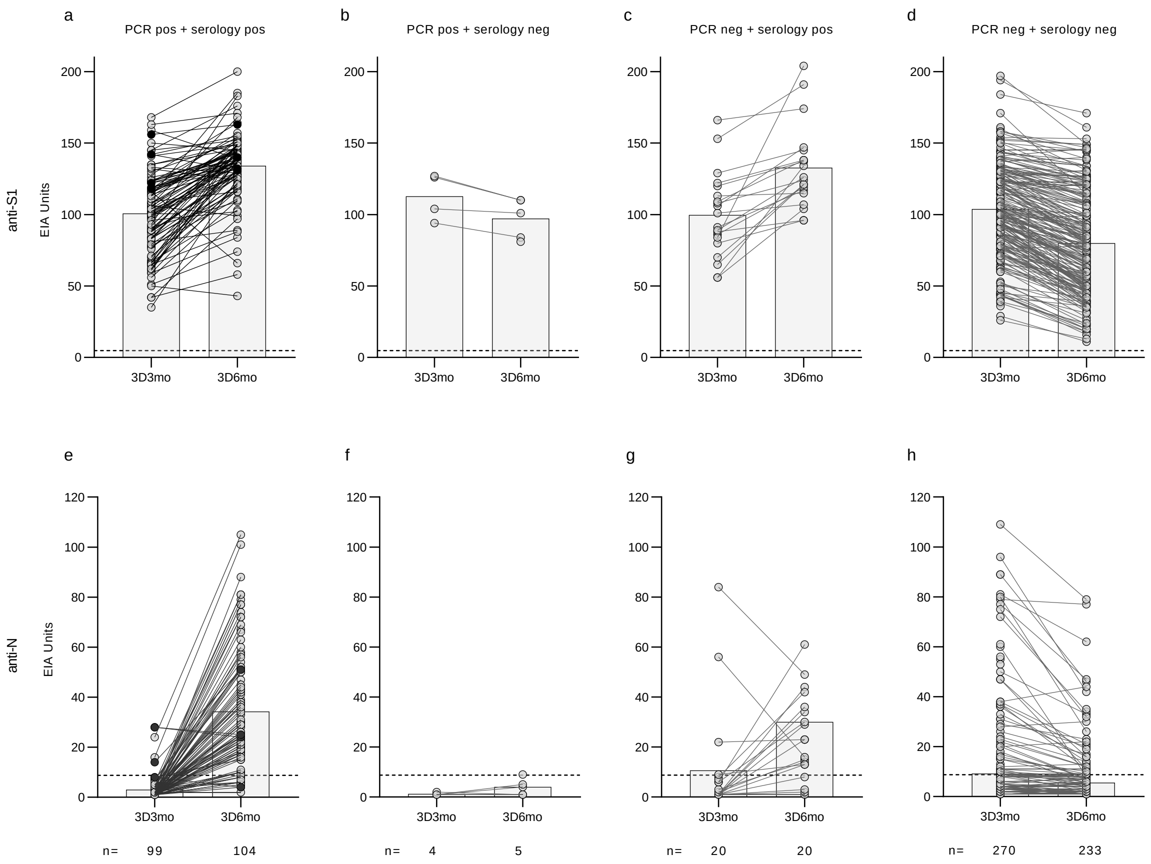

Supplementary figure 2. Changes in SARS-CoV-2 anti-S1 and anti-N antibodies from three months to six months post third vaccine dose grouped by PCR test or antigen test positivity and serologically confirmed infection. HCWs were grouped into four groups (PCR pos/neg and serology pos/neg) based on their PCR or antigen test positivity three months to six months post third vaccine dose. Four vaccinees with a PCR positive test between three weeks and three months after third dose and increase in antibodies between three and six months after third dose were included in the PCR pos –group. Prevaccination infected vaccinees were marked with black dots. SARS-CoV-2 **a-d** S1-specific and **e-h** N-specific antibody responses of each group and the number of samples in each time point is indicated.

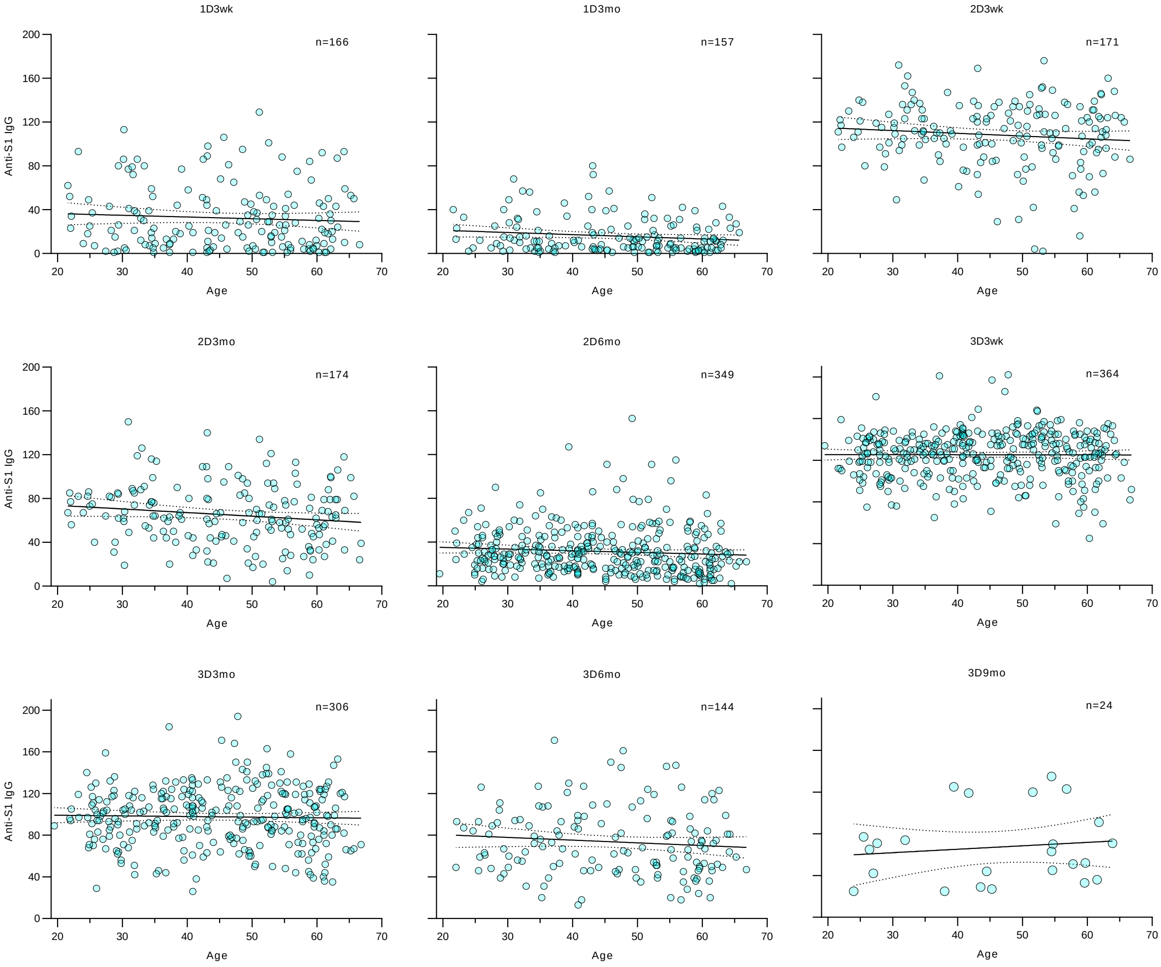

Supplementary figure 3. Vaccine-induced SARS-CoV-2 anti-S1 IgG antibody levels in relation to age in HCWs following one, two, or three COVID-19 vaccine doses. SARS-CoV-2 anti-S1 IgG antibody levels of uninfected HCWs were plotted according to the age of each HCW. The antibody levels in different time points after one, two, and three COVID-19 vaccine doses were analyzed. The number of samples in each time point as well as a linear regression line with a 95% confidence interval (dotted lines) are shown.

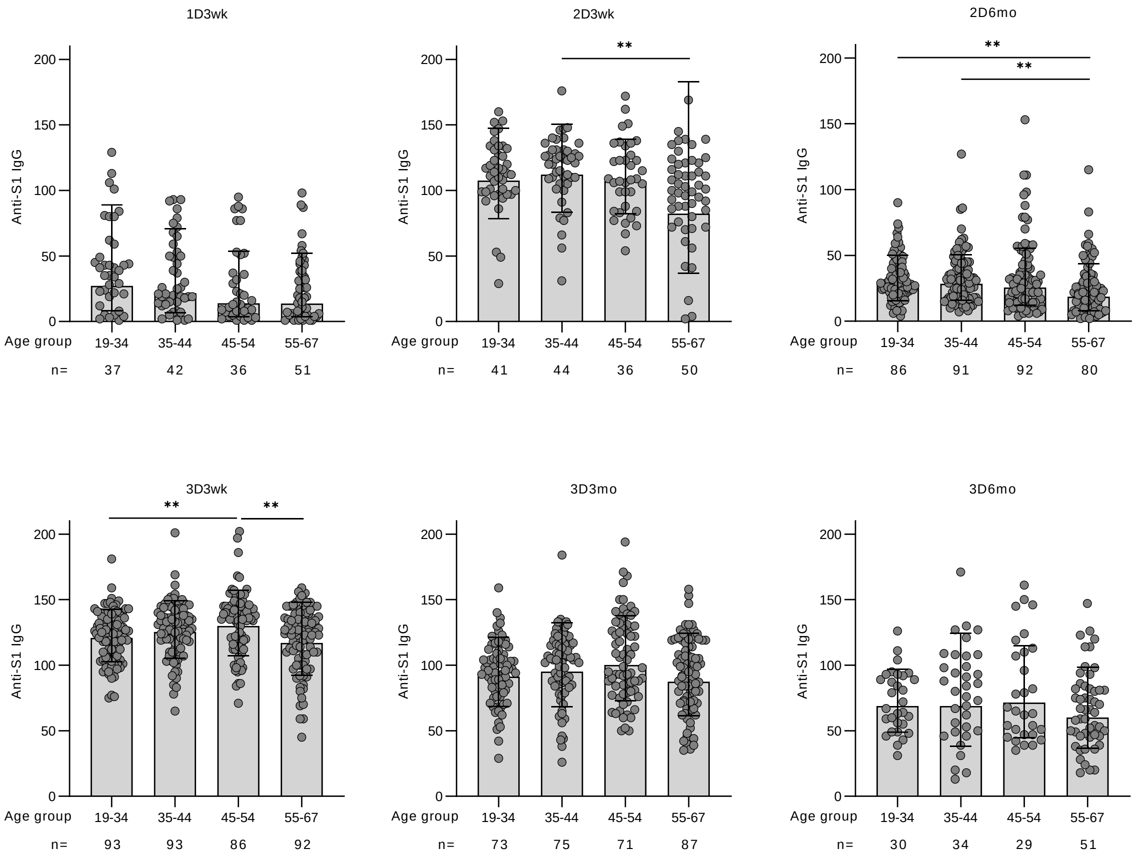

Supplementary figure 4. Age-stratified SARS-CoV-2 anti-S1 IgG antibody levels in HCWs following one to three COVID-19 vaccine doses. SARS-CoV-2 anti-S1 IgG antibody levels of uninfected HCWs were stratified by age. Number of samples for each group is indicated. Geometric means with standard deviations are shown as bars and lines.

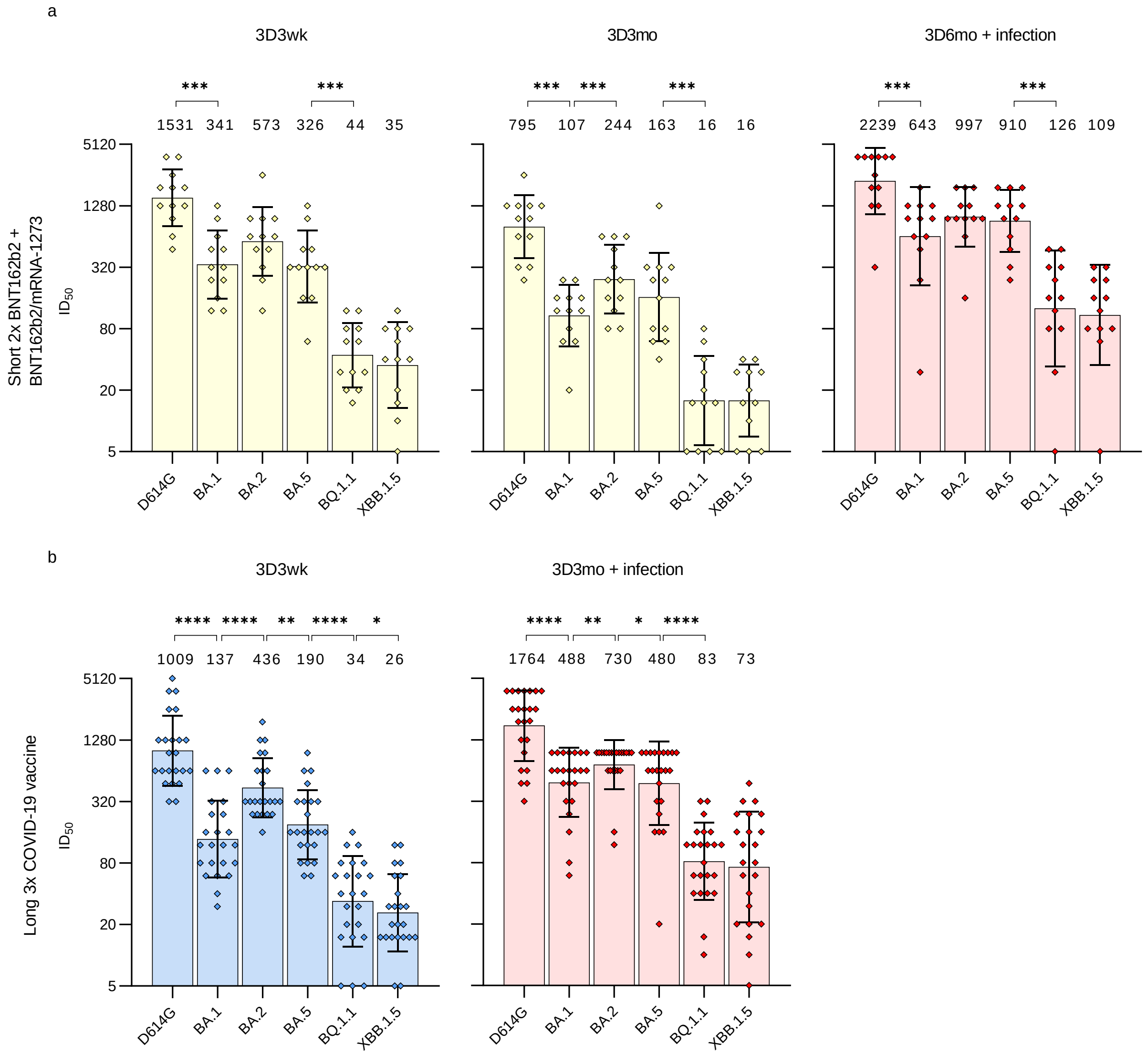

Supplementary figure 5. Neutralization of six SARS-CoV-2 variants after three vaccine doses and a SARS-CoV-2 infection. Neutralizing antibodies against D614G and Omicron variants BA.1, BA.2, BA.5, BQ.1.1, and XBB.1.5 of **a** 12 HCWs with short vaccination interval and **b** 23 HCWs with long vaccination interval with a breakthrough infection were compared. Half-maximal inhibitory dilutions (ID_{50}) were calculated three weeks (3D3wk), three months (3D3mo), and six months (3D6mo) after receiving the third COVID-19 vaccine dose. Titers <10 were marked as 5. Geometric mean antibody titers with standard deviations are shown as a line and value for each variant.

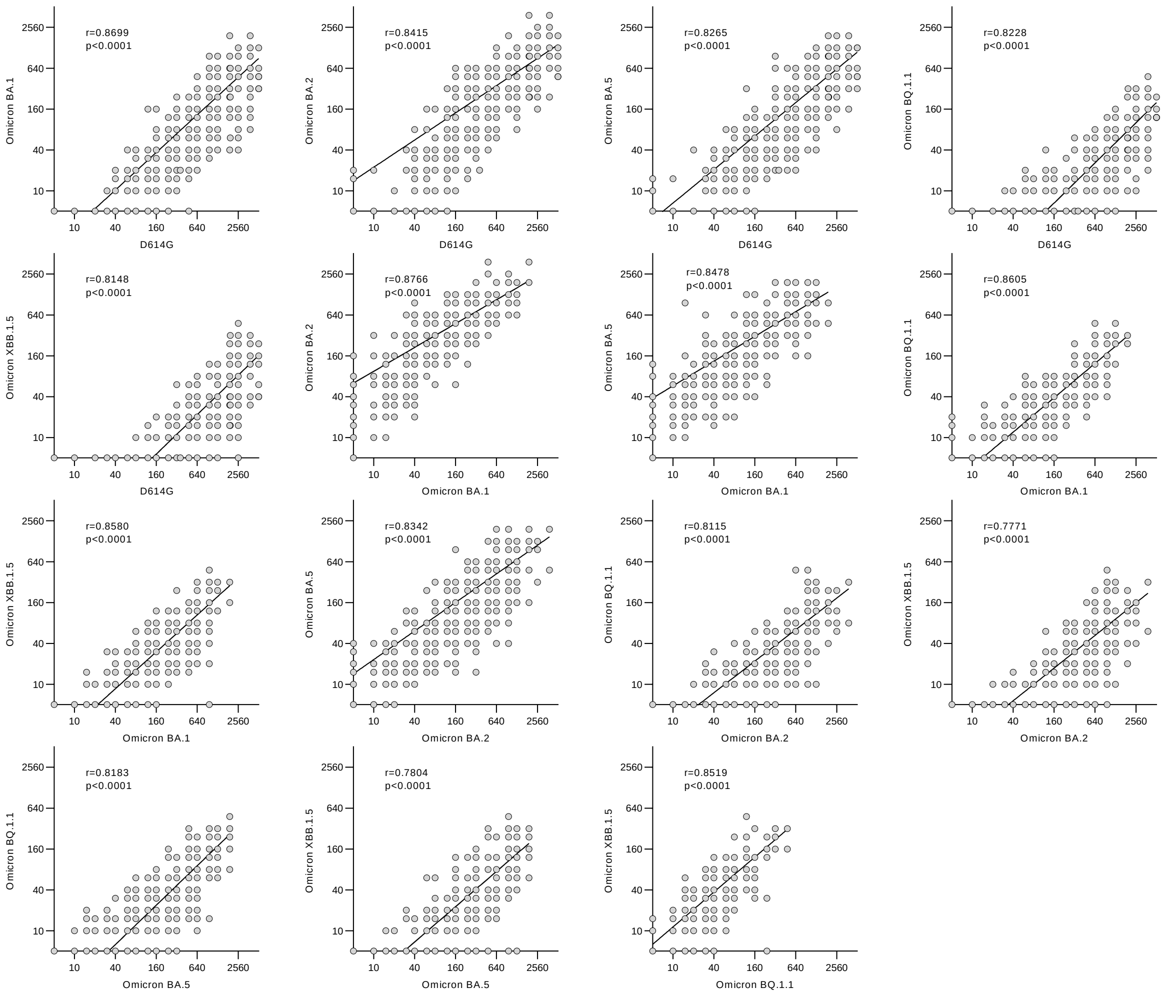

Supplementary figure 6. Correlation of neutralizing antibody responses against SARS-CoV-2 D614G and five Omicron variants. Altogether 499 serum samples collected from 155 vaccinees after two or three COVID-19 vaccinations were analyzed for neutralizing antibodies for SARS-CoV-2 D614G and Omicron variants BA.1, BA.2, BA.5, BQ.1.1, and XBB.1.5. Pairwise correlation of neutralization titers for the six variants were analyzed with Spearman's rank order correlation. The correlation coefficient (r), two-tailed p-values, and regression line corrected for logarithmic axis are shown for each variant pair.

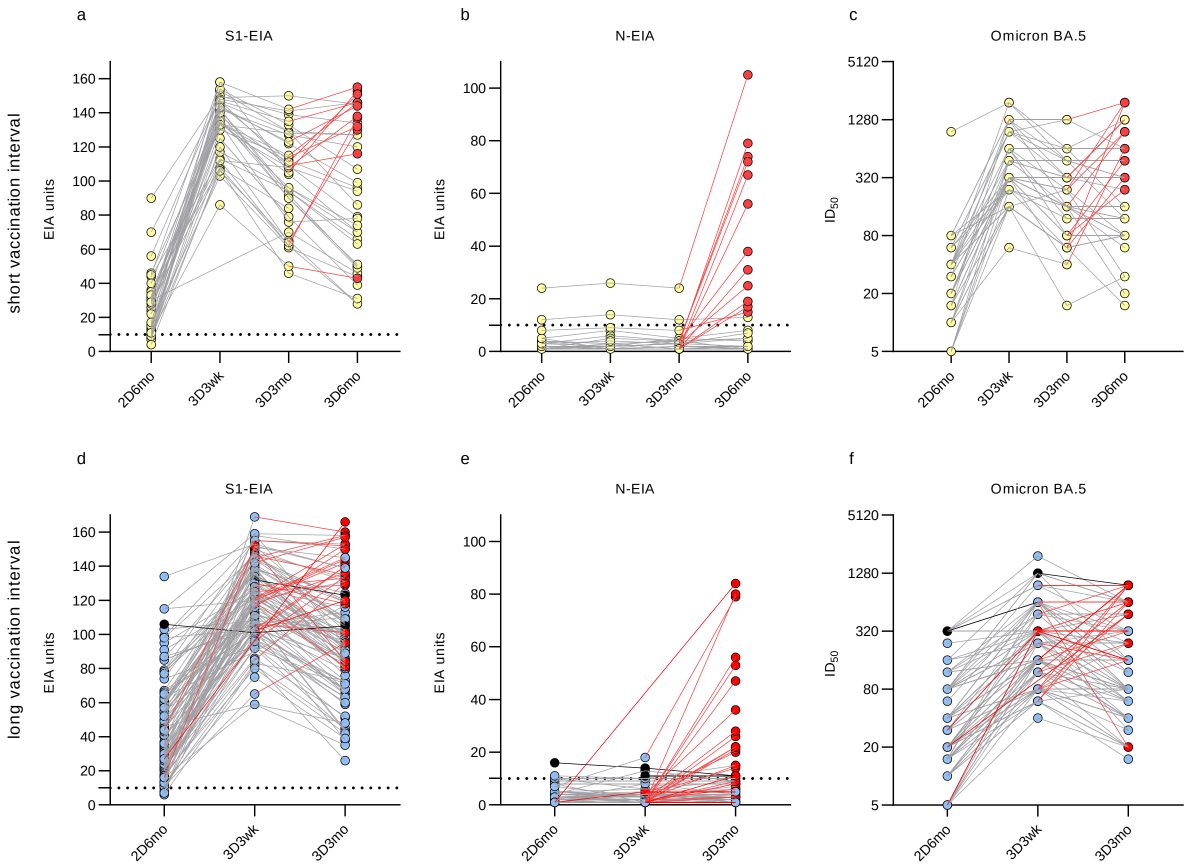

Supplementary figure 7. Congruence of antibody responses detected by SARS-CoV-2 S1-EIA, N-EIA, and Omicron BA.5 MNT. a-c Sera of 41 HCWs with a short vaccination interval and **d-f** 114 HCWs with a long vaccination interval were measured for SARS-CoV-2 S1-specific and N-specific IgG antibodies by EIA and for Omicron BA.5 neutralizing antibodies by MNT before (2d6mo, n=41 and 112) and after (3D3wk, n=40 and 112; 3D3mo, n=41 and 114; 3D6mo, n=39) the third COVID-19 vaccination with BNT162b2 or mRNA-1273. Grey lines connect the antibody levels measured and half-maximal inhibitory dilutions (ID_{50}) of sequential serum samples of each vaccinee. Red lines indicate intervals between time points where a vaccinee has had a SARS-CoV-2 infection (PCR-, antigen test -, or serologically confirmed) and red dots the antibody levels or titers after the infection. Two individuals (black dots connected with black lines in panels **d-f**) had a SARS-CoV-2 infection before vaccinations.
