# Supplementary material for: Neutralizing antibodies after the third COVID-19 vaccination in healthcare workers with or without breakthrough infection
Source: Commun Med (Lond). 2024 Feb 23;4:28. doi: 10.1038/s43856-024-00457-3 (PMC10891120; doi:10.1038/s43856-024-00457-3)
Supplement: Supplementary file 3 — Description of Additional Supplementary Files [file 43856_2024_457_MOESM3_ESM.pdf]

**Description of Additional Supplementary Files**

**File name:** Supplementary Data 1

**Description**: Source data for for main figures and supplementary figures.
